# The Keys to Unleashing Potential

**DOI:** 10.21470/1678-9741-2021-0961

**Published:** 2021

**Authors:** Glen Van Arsdell

**Affiliations:** 1 Department of Congenital Heart Surgery, University of California, Los Angeles, United States of America

In the past issue of Brazilian Journal of Cardiovascular Surgery, Miana LA et al.^[1]^ have transparently demonstrated technical performance scores (TPS Classes 1 to 3) for 972 congenital cardiac surgical cases. A TPS Class 3 (inadequate) was independently associated with higher mortality and morbidity. A higher TPS score was co-linear with longer bypass times and complexity. In other words, it is harder to do harder surgery. The authors make an interesting observation that even with TPS scores of 1 (optimal) and 2 (adequate), observed mortality was higher than that reported in some developed countries^[2]^.

I applaud this type of data analysis. It allows for feedback, and thus effortful learning for performance enhancement. While operative quality is crucial, it is not the only crucial component to best outcomes. For that, a spectrum of things need to be optimal: diagnosis, timing of repair, operating room capabilities beyond surgical performance, postoperative care, and the potential need for postoperative diagnosis and intervention. In short, it is optimal team performance of multiple disciplines^[3,4]^.

Globally, there can be differences in resources, timing of patient presentation, and experience of practitioners. Human talent and human nature are not different. The question then becomes: how does one create an environment for talent to achieve its potential?

I believe it is about culture, history, and incentives. Key components of an optimal culture are data (observational and collated data), continuous multidisciplinary team learning from that data (with learnings implemented and outcomes remeasured), and intellectual honesty around that data and learning. It should be done in a supportive environment. In other words, deal with the data honestly but also respectfully. That can be hard. With regard to human nature, historical organizational design of separate surgery, pediatrics, anesthesia, imaging, and even intensive care units tend to make for malalignment of incentives to work together. This must be overcome either functionally or structurally. This can also be difficult. Optimal outcomes occur when it is always about delivery of best care to the patient and not about protecting lines of authority or incentives. In fact, those last items are far too often detrimental. These types of detrimental challenges are seen throughout the world. Those programs that best manage to learn from data, act on it regardless of what the data says, and do so as a cohesive team, will over time, perform at the highest level possible for that particular environment - developed or developing program or nation.

A brilliant surgeon, cardiologist, intensivist, imager, and anesthesiologist in Brazil is brilliant in North America or any country. It is not enough. We must work together to collect, process, and learn from data inclusive of the entire patient course - diagnostics, operative care, and postoperative care. Continuous, intellectually honest learning and implementation of that learning as a team will unleash the potential of any given institution. It is best for the babies and children. It is truly a moral obligation.
